# Demystifying machine learning for mortality prediction

**DOI:** 10.1186/s13054-021-03868-z

**Published:** 2021-12-23

**Authors:** J. M. Smit, M. E. van Genderen, M. J. T. Reinders, D. A. M. P. J. Gommers, J. H. Krijthe, J. Van Bommel

**Affiliations:** 1 Department of Intensive Care, Erasmus University Medical Center, Rotterdam, Netherlands; 2 EEMCS, Pattern Recognition and Bio-informatics Group, Delft University of Technology, Delft, Netherlands

With interest, we read the article by Banoei et al. [1] on machine learning (ML) models to predict mortality among COVID-19 patients. They refer to other studies that failed to predict mortality using ‘conventional statistical analysis’, after which they present a linear ML model as a better suited method for such complex medical problems. We feel such a claim creates an image around ML as an alternative technique that offers solutions where statistical modeling fails. However, ML and statistical modeling are tightly interwoven. Indeed, there is no consensus on whether or how to differentiate between the two [2, 3], e.g., the approach Banoei and colleagues present (Partial Least Squares) could easily be considered a ‘statistical analysis’ as well. A recent systematic review [4] differentiates ML from statistical models based on how ‘automatically’ these models learn and found no difference in discriminative performance. This raises the question why efforts are made to differentiate between statistical modeling and ML, as it does not provide insights into which prediction models work for which kind of problems. We advocate it is more important to demystify ML by emphasizing its connections to statistical models most clinicians are already familiar with, as it may help in setting reasonable expectations for the potential clinical benefit ML could bring. Towards demystifying ML, good reporting of methodology and findings is essential. Due to unclear or incomplete reporting, one may draw wrong conclusions and miss out on opportunities to learn. For instance, Banoei and colleagues are unclear on how frequently measured predictors (like SpO2) were aggregated to one value. We encourage to follow a unified approach for reporting, e.g. the TRIPOD guidelines [5]. Moreover, it is unfortunately still common that the intended use of prediction models is unclear, whereas it has implications for choices in the model development. Likewise, Banoei and colleagues present several models (for mortality prediction and patient clustering) and suggest that these can ‘aid in clinical decision making and resource allocation’, without further specification. We strongly encourage to develop models with an explicit intended use in mind, enabling fair judgement of their clinical relevance. Altogether, we join Banoei and colleagues in their belief that ML models hold a lot of promise as valuable tools in the modern ICU. However, we advise against differentiating them from statistical models and advocate proper reporting about the methodology and intended use.

## Abbreviations

ML: Machine learning
ICU: Intensive care unit
COVID-19: Coronavirus disease 2019
TRIPOD: Transparent Reporting of a multivariable prediction model for Individual Prognosis or Diagnosis
