# Supplementary material for: Demystifying machine learning for mortality prediction
Source: Crit Care. 2021 Dec 23;25:447. doi: 10.1186/s13054-021-03868-z (PMC8697544; doi:10.1186/s13054-021-03868-z)
Supplement: Related texts

# Reply: Machine learning-based methods vs. conventional statistical methods for studying mortality outcome

**Authors:** Mohammad M. Banoei, Roshan Dinparastisaleh, Ali Vaeli Zadeh, Mehdi Mirsaeidi

**Affiliations:** 3 grid.22072.35 0000 0004 1936 7697 Department of Critical Care Medicine, University of Calgary, Alberta, Canada; 4 grid.22072.35 0000 0004 1936 7697 Department of Biological Science, University of Calgary, Alberta, Canada; 5 grid.21107.35 0000 0001 2171 9311 Division of Pulmonary and Critical Care Medicine, Johns Hopkins University, Baltimore, MD 21218 USA; 6 grid.484420.e Division of Pulmonary and Critical Care, Miami VA Medical Center, Miami, FL USA; 7 grid.15276.37 0000 0004 1936 8091 Division of Pulmonary and Critical Care and Sleep Medicine, Department of Medicine, College of Medicine, University of Florida, Jacksonville, FL USA

In response to the Letter to the Editor by J.M. Smit et al., we certainly agree that machine learning (ML)-based methods can be used interchangeably with multivariate data analysis (MVA) and multivariate prediction models (MPMs) [6]. Although ML approaches like partial least square (PLS), statistically inspired modification of PLS (SIMPLS), random forest (RF), Support Vector Machine (SVM), and artificial neural network (ANN) are considered statistical methods, they are notably different from conventional statistical methods (CSM) [7]. Considering the advantages of ML methods, ML approaches have contributed significantly to the early detection, tracing, diagnosis, prognosis and clinical trials of COVID-19 that have been more functional to support researchers in confronting the coronavirus pandemic [8]. Several studies previously have shown that ML can be more appropriate than CSM for the clinical datasets. ML algorithms have proven better able to stratify COVID-19 patients and mortality risk [9], identify high-risk patients with COVID-19 [10].

It is generally believed that there is no single ML method superior to others. The SIMPLS method was the only prediction model used in our current study. SIMPLS has remarkable advantages including a lower risk of overfitting, a high level of interpretation, a high level of variable selection, easy implementation when compared with RF, SVM and ANN methods [6]. SIMPLS can easily account for batch processing and a high degree of correlation (multicollinearity) between and among variables of large datasets. SIMPLS fit the outcome responses with nominal, continuous, and polynomial data type, interaction and categorial effects, and provides strong visualization when compared with other ML and CSM. In our study, SIMPLS was successfully applied to recognize the most differentiating variables involved in the prediction of COVID-19 mortality. Other statistical methods that are not considered prediction models, such as principal component analysis (PCA) and latent class analysis (LCA) were established based on the findings of SIMPLS to identify patients at the highest risk of dying.

In our study, the importance of ML-based model was its ability to predict patient mortality using variables measured at the time of admission, although these variables were frequently measured during the patients’ hospitalization for other purposes.

Our study mainly focused on the importance of ML in clinical application, and the recognition of the most important variables contributing to mortality from COVID-19, instead of describing the details of ML that may be extraneous for clinicians.
